# The Trends and Risk Factors of AIDS-Defining Cancers and Non-AIDS-Defining Cancers in Adults Living with and without HIV: A Narrative Review

**DOI:** 10.1155/2024/7588928

**Published:** 2024-03-21

**Authors:** Anikie Mathoma, Benn Sartorius, Saajida Mahomed

**Affiliations:** ^1^College of Health Sciences, University of KwaZulu-Natal, Durban, South Africa; ^2^University of Botswana, Gaborone, Botswana; ^3^Faculty of Medicine, University of Queensland, Brisbane, Australia; ^4^Centre for Tropical Medicine and Global Health, University of Oxford, Oxford, UK; ^5^Department of Health Metric Sciences, University of Washington, Seattle, USA

## Abstract

**Background:**

People living with HIV (PLHIV) are at a high-risk of developing AIDS-defining cancers (ADCs) and non-AIDS-defining cancers (NADCs). This review is aimed at exploring available evidence regarding the trends of ADCs and NADCs and the associated risk factors among adult PLHIV.

**Methods:**

We conducted a comprehensive search of PubMed, Web of Science, and EBSCO host databases to identify articles published between 2010 and 2023 that reported incidence and mortality rates of cancer, including ADCs and NADCs among PLHIV. We compared trends and rates in PLHIV with HIV-negative adults and further assessed related risk factors.

**Results:**

A total of 1886 potentially eligible articles were screened, and of these, 36 were included in this study. More than 50% (*n* = 20) of these were based in high-income countries. Seventeen studies reported a higher prevalence of NADCs compared to ADCs, with twelve of these conducted in high-income countries. Conversely, eight out of twelve studies reporting a higher prevalence of ADCs versus NADCs were from low-and-middle and upper-middle-income countries. Ten studies indicated a higher incidence of ADCs (6 studies) and NADCs (4 studies) among PLHIV compared to HIV-negative individuals. In contrast, only two studies observed an increase in NADCs among the HIV-negative population. In comparing mortality, seven out of nine studies showed elevated NADC-related deaths compared to ADCs. The main risk factors identified for any cancer, NADCs, and related mortality were advancing age, and longer duration of HIV infection, while lower CD4 cell counts (<200 cells/*μ*l), was associated with both ADC and NADC occurrences.

**Conclusion:**

Chronic HIV infection combined with advancing age in PLHIV taking antiretroviral therapy appears to have contributed to increasing cancer burden, particularly the incidence of NADCs and associated mortality. These findings stress the importance of screening for high-risk cancers among PLHIV for early detection and treatment to ensure improved outcomes.

## 1. Introduction

According to the World Health Organization (WHO), the human immunodeficiency virus (HIV)/acquired immunodeficiency syndrome (AIDS) epidemic continues to be a public health threat with an estimated 38.4 million people living with the virus globally in 2021, and of these, nearly 70% (25.6 million) reside in the African region [1]. People living with HIV (PLHIV) have an elevated risk of developing many opportunistic infections and cancers, primarily due to HIV-related immunosuppression, which impairs the control of oncogenic viral infections [2, 3]. The WHO estimates that as of 2020, cancer was the leading cause of mortality worldwide accounting for almost 10 million deaths with lung cancer leading with 1.8 million deaths [4]. Stomach, breast, colon, esophagus, pancreas, lung, and prostate versus one virus-related cancer (liver cancer) were leading in the number of cancer-related deaths by the end of 2020 [5].

Cervical cancer, Kaposi sarcoma (KS), and non-Hodgkin lymphoma (NHL) are the most common cancers among PLHIV [6–11], and these are classically referred to as AIDS-defining cancers (ADCs) as they indicate clinically relevant immunosuppression [2]. The introduction and expansion of combined antiretroviral therapy (cART) in the past two decades has resulted in a decline in the incidence of these cancers in many countries especially the high-income countries (HICs) [12–16]. Cervical cancer, KS, and NHL all have an infectious etiology: KS is caused by human herpesvirus 8 (HHV-8) [17], cervical cancer is caused by human papilloma virus (HPV) [18], and NHL is linked to Epstein-Barr virus (EBV) [19]. The risk of these ADCs remains high in many low- and middle-income countries (LMICs) where there are significant HIV-infected populations who are not on treatment or who are on treatment but not virologically suppressed [20–23]. Data from recent studies conducted in the United States of America (USA), Europe, and Australia have shown continued declines in the rates of ADCs [24–27]. Nevertheless, when compared with the general population, a USA study noted that while there has been dramatic declines in the trends of ADCs over the years, the rates of KS, NHL, and cervical cancer in PLHIV remain elevated at approximately 800-fold, 10-fold, and 4-fold, respectively, when compared to their HIV-negative counterparts [24]. According to the International Agency for Research on Cancer (IARC), LMIC regions had the highest cancer-related mortality rates caused by ADCs worldwide in 2020. Cervical cancer, NHL, and KS accounted for the highest cancer-related deaths in Africa, with cervical cancer causing 10.8% of deaths, NHL causing 4.4%, and KS causing 1.8%. In Asia, cervical cancer accounted for 3.4% of deaths, while NHL caused 2.3% of deaths [5].

As stated above, the effectiveness of cART in reducing ADCs burden has been demonstrated; however, there has been emergence of other cancers like lung, nonmelanoma skin, hepatocellular cancer (HCC), anal, and oropharyngeal categorized as non-AIDS-defining cancers (NADCs), and these type of cancers seem to occur more frequently in PLHIV who have been on ART and virologically suppressed for a long period [14, 27–33]. While the carcinogenic or anticarcinogenic potential of cART in NADCs is yet to be established, effective cART is thought to be fueling the incidence of NADCs because of its ability to increase survival and subsequently lead to prolonged life and time with HIV infection [29]. Non-AIDS-defining cancers can be classified into virus-related and virus unrelated. Examples of the virally mediated cancers are liver cancer caused by the hepatitis B and C viruses (HBV and HCV); vulva, penis, anal, oropharynx, and larynx cancers caused by HPV; and Hodgkin's lymphoma caused by EBV. Lung, breast, stomach, and prostate cancers are some of the NADCs without a link to an underlying coinfectious agent [14, 34–36]. In addition to the infectious agents linked to ADCs and NADCs, demographic factors such as age; behavioral risk factors such as smoking, alcohol use, unhealthy diet, and physical inactivity; and environmental factors such as air pollution have also been identified as important drivers of cancer [4]. Most of these risk factors are attributed to a rise in NADCs such as liver, lung, and esophagus.

In response to the growing burden of cancer, the WHO has adopted the 2030 United Nations Agenda for Sustainable Development to reduce premature mortality from cancer by (i) monitoring the cancer burden through cancer registries and (ii) developing standards and tools to guide the planning and implementation of interventions for prevention, early diagnosis, screening, treatment, and palliative as well as survivorship care [4]. This narrative review is therefore aimed at (i) reviewing trends of ADCs and NADCs and the evolving risk factors as important indicators to show the progress made in reducing cancer incidence and mortality for individual cancer types and for cancer overall and (ii) identifying cancers that may be increasing in incidence. The data may likely improve our understanding of the trend assumptions, and underlying factors attributed to cancers that are increasing in incidence among PLHIV which may highlight a greater need for expanded access to HIV therapies, cancer prevention, screening, and treatment. Additionally, the data may suggest the need to assess the applicability of the current screening guidelines for PLHIV.

## 2. Methods

### 2.1. Search Strategy

Online databases including PubMed, Web of Science, and EBSCO host were searched for peer-reviewed research articles published on cancer trends and associated risk factors in English regardless of study setting or geographical area. The search was restricted to studies published from January 1, 2010, to December 31, 2023, to better understand the cancer trends in the second decade of ART roll-out. This period has been characterized by an increase in access to ART and the introduction of universal ART by the WHO in 2016 [37]. The following keywords were used on the search databases: cancer, cancer trends, HIV, AIDS-defining cancers, non-AIDS-defining cancers, incidence, prevalence, mortality, and risk-factors. Medical Subject Heading (MeSH) terms were used to search for the cancer incidence and mortality as well as specific cancer risk factors including smoking, alcohol consumption, overweight or obesity, HBV (“hepatitis B”), HPV (“papillomavirus infections” or “Papillomaviridae”), EBV (“Epstein-Barr virus infections”), and HHV8 (“herpesvirus 8, human”).

### 2.2. Eligibility Criteria

The following inclusion criteria were applied:

- Involving individuals aged 18 years and above
- Conducted between 2010 and 2023
- Using quantitative study designs
- Published in English language only

The following exclusion criteria were applied:

- Involving children; or
- Published before 2010
- Using qualitative methodology
- Published in language other than English

### 2.3. Data Extraction

Data were extracted and collated in Microsoft Excel. Quantitative studies including cross-sectional, retrospective, and prospective cohort studies were identified. While cross-sectional data cannot estimate cancer incidence and mortality rates, the studies can be useful in estimating prevalence and identifying trends because of the ability to collect data across different groups at a given point in time [38, 39]. The extracted data from each article included the authors' names, year of publication, the date/period of the study, and the study design. Other information extracted were the setting/country(ies) where the study was conducted, the study population/sample, sex/gender studied and key findings presented as proportions/incidence/mortality rates of cancers, ADCs and NADCs, and the measurement of various risk factors.

### 2.4. Data Analysis

Articles were tabulated and summarized by any cancer, ADCs (KS, NHL, and cervical cancer) and NADCs. Where applicable, NADCs were categorized as virus-related and nonvirus-related and compared among the PLHIV and those without HIV. Burden trends for individual ADCs and NADCs were assessed to identify increases or decreases in incidence over different time periods from pre-ART through late cART era. Trends between individual LMICs and HICs were further summarized and compared. Risk factors for cancer incidence and mortality were also summarized.

## 3. Results

**Figure 1:**
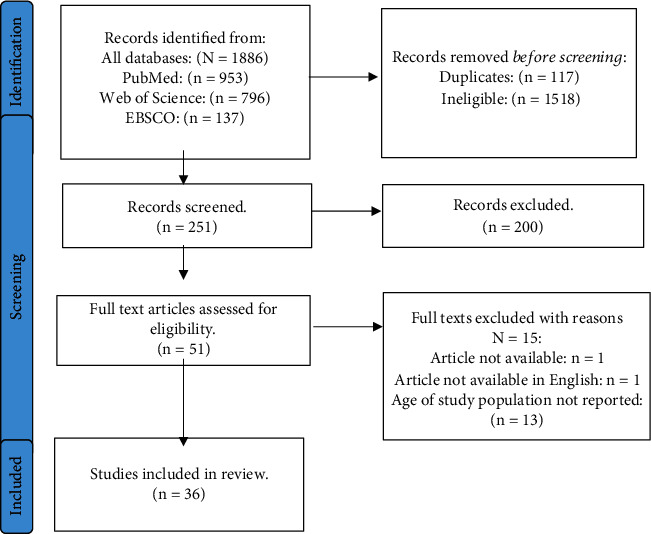
PRISMA flow diagram showing literature search of databases and studies included.

A total of 1886 articles were identified from the three databases. Applying the criteria, 1635 studies were excluded (Figure 1). Each of the remaining 251 abstracts was screened for eligibility, and 51 were included for full text article review. Out of these, 36 studies were deemed eligible for inclusion in the analysis. The characteristics of the included studies are outlined in Table 1. Majority of the studies, 22 (61%) used a retrospective cohort study design and seven (19%) were cross-sectional. More than 50% (*n* = 22) of the studies were carried out in HICs, six (17%) were done in the LMICs, and four (11%) in low-income countries (LICs), specifically in sub-Saharan Africa (SSA). Out of the 28 studies that specified gender, 22 (79%) had a sample that constituted more males than females, 4 (14%) had more females than males, and all of them were in SSA, and for each of the remaining two studies, one focused on males and the other on females only. Out of the 36 studies, 31 assessed the incidence of both ADCs and NADCs, and 15 of these reported on mortality. Five studies focused on NADCs only.

The leading individual ADCs were NHL and KS reported by 11 studies each, and majority of these studies were from upper-middle and high-income countries. Cervical cancer was reported by seven studies as the most common ADCs, and five of these studies were done in SSA. The leading NADC reported by nine studies was lung cancer followed by HL and breast cancers. Similarly, all the five out of seven studies that reported breast cancer as the commonest were from LMICs based in SSA.

Table 2 shows 31 of the 36 studies that compared the incidence and mortality of ADCs and NADCs. A total of 17 studies found higher incidence of NADCs when contrasted with ADCs, and 12 of these were conducted in HICs. Twelve studies reported higher incidence of ADCs than NADCs, and of these eight were from LMIC and UMICs. Only two studies showed similar proportions of both ADCs and NADCs. Nine studies compared cancer incidence between the PLHIV and the HIV negative or general population, and of these, six studies from both HICs and LMICs countries reported a higher incidence of ADCs in PLHIV compared to their HIV-negative counterparts. Similarly, four studies showed an increase of NADCs in PLHIV versus those HIV negative. Only two studies observed an increased rate of NADCs including nonvirus-related NADCs in the HIV-negative individuals, while one study showed similar rates of NADCs in both groups.

A total of 11 studies compared cancer-related mortality between ADCs and NADCs, and of these, seven studies from HIC and UMICs reported more NADC-related deaths than ADC-related deaths, three studies reported more ADC than NADC-related deaths, while only one study revealed similar proportions of ADC and NADC-related deaths. When compared to the general population, only two studies, one from a HIC and the other from a LIC, revealed higher rates of overall cancer mortality among PLHIV.

Four studies compared the incidence of virus and nonvirus-related NADCs among PLHIV. Of these, only one study from Italy found an increase in virus-related NADCS versus nonvirus ones, while the remaining three (all of them done in USA) showed that nonvirus-related NADCs were in the increase when compared with virus-related NADCs. Mortality was investigated by the Italian study only, and the results showed more nonvirus NADC-related deaths versus virus-related NADC deaths.

A total of seven studies (6 HICs and 1 UMIC) documented a decline in the incidence ADCs and NADCs during ART program expansion. In contrast, three studies (2 HICs and 1 UMIC) reported an increase in NADC rates while four others showed that the incidence of NADCs remained stable over the ART expansion period. Decreasing overall cancer rates were noted by only two studies from HICs.

Risk factors associated with cancer incidence and mortality are summarized in Table 3. The most common factor identified as a risk for the occurrence of any cancer was advancing age reported by three studies, followed by low CD4 counts < 200 cell/*μ*l documented by two studies. Other important risk factors for cancer among PLHIV were longer duration of HIV infection, coexistence of clinical AIDS, tobacco use, male gender, and not being on ART, each noted by one study. Five studies also identified advancing age as a significant risk factor for non-AIDS-defining cancers (NADCs), aligning with the aforementioned factors. Only three studies evaluated the risks linked to ADCs with the main risk factor being low CD4 count < 200 cell/*μ*l at ART initiation. Other risks for ADCs reported by one study each were lack of viral load suppression, presence of opportunistic infections, and previous cancer history.

Factors associated with increased risk of any cancer-related mortality among PLHIV were identified by one study as poor immune status, lack of cancer treatment, and cancer staging III and IV. Other predictors of cancer mortality were high viral load > 400 copies/ml reported by two studies, increasing age documented by two studies, longer duration with HIV noted by one study, and being not on ART also reported by one study. Specifically, deaths related to NADCs were linked to advancing age, CD4 count < 200 cell/*μ*l, and AIDS comorbidity documented by two studies each. Lower CD4 count < 200 cell/*μ*l was further identified by two studies as a high risk for ADC-related mortality.

## 4. Discussion

This narrative review provides an overview of the trends of ADCs and NADCs in PLHIV compared to the general population. In more than half of the studies examined, elevated numbers of NADCs were observed in PLHIV when compared to ADCs. The review also revealed a declining trend in ADCs [50, 58, 61, 65, 69], while NADCs showed an increasing [57, 58, 65] or stable pattern [32, 50, 61, 69] from the pre-ART to late ART period. These findings align with previous studies indicating that the introduction and expansion of cART over the past two decades has significantly improved immune function and life expectancy among PLHIV, leading to a decrease in ADC incidence and an increase in NADCs due to prolonged survival [42, 53, 73]. This pattern was further confirmed by four studies [58, 67, 69, 71] in our review that compared virus-related NADCs to nonvirus-related NADCs in PLHIV, and three of these studies all from USA found that the latter were on the rise. As with ADCs, virus-related NADCs are linked to similar infectious agents such as HPV and the declines in their incidence could also be attributed to the effectiveness of ART. However, there is a need for research to understand the risk and trends of this class of NADCs particularly in LMICs. None of the LMIC studies in our review investigated NADCs as virus or nonvirus related.

Despite decreasing rates of ADCs, this review found that when compared with the HIV-negative individuals, all relevant studies documented higher ADCs rates among PLHIV. The most prevalent ADCs were NHL and KS in the upper-middle and high-income countries and cervical cancer in LMICs. Regarding NADCs, a similar trend was seen where seven of the nine studies reported increased rates of NADCs in PLHIV with lung cancer taking the lead. This observation is supported by data from SSA countries that have demonstrated that cancer associated with HIV/AIDS is a prevalent problem despite the roll out of cART where PLHIV were found to be at higher risk of ADCs than the general population [6–11, 74]. Therefore, with the rates of ADCs and NADCs elevated in PLHIV, it is paramount that healthcare providers prioritize regular screening and early detection, promote health education and health lifestyle behaviors, and offer comprehensive management for ADCs and NADCs in this population, while sustaining access to and adherence to cART.

Our analyses by country-income status showed that the overall incidence of ADCs in PLHIV were higher in LMICs while NADCs were higher in HICs. The increased rates of NADCs in HICs are consistent with evidence from several studies that have noted that while the scale-up of cART in HICs has led to significant declines in ADCs, the incidence of NADCs has been increasing [31, 32, 36, 75]. On the other hand, the trend of higher ADCs in LMICs has been attributed to various reasons including a substantial number of HIV-infected people who are not aware of their HIV status, with some initiating ART late with low CD4 cell counts < 200 cell/*μ*l and advanced immunosuppression while others are not on ART despite being eligible for the treatment [1, 76]. In our review, cervical cancer, an ADC, was the most prevalent cancer in LMICs with all the studies from SSA reporting it as the leading cancer cause.

The majority of studies that reported on mortality in this review showed that when compared with ADCs, NADCs were responsible for more cancer-related deaths in PLHIV with lung cancer being the leading cause. The incidence of lung cancer has been found to be substantially higher in PLHIV, partly due to aging and tobacco use and also because of immunosuppression and inflammatory processes associated with chronic HIV infection [60, 77, 78]. Another study in this review found that women with cancer and HIV-infection with lower CD4 cell counts had shorter survival when compared with their HIV-negative counterparts [49]. The high cancer-related mortality among PLHIV is also linked to late presentation with advanced disease [79] with six studies [40, 41, 55, 60, 68, 71] in our review reporting that the majority of PLHIV with cancer presented with stages III and IV or advanced disease.

The most common risk factors associated with the occurrence of any cancer, NADCs, and the related cancer mortality were advancing age [46, 52, 54, 57, 59, 61, 63, 67, 72] and longer duration with HIV infection [11, 52, 54, 56, 64, 72]. A US-based study conducted over a 15-year period has shown that increases in the rates of NADCs were mainly driven by growth and aging of PLHIV [29]. Our review further revealed that lower CD4 cell counts < 200 cell/*μ*l [32, 47, 59, 67, 69, 70, 72] was also noted as a prevalent risk for ADCs, NADCs, and associated mortality highlighting the role that immunosuppression plays in predisposing PLHIV to cancer. Another important risk factor for cancer incidence and mortality observed in this review was male gender. Though reported by only three studies [45, 59], this finding is not surprising as we noted that two-thirds of all the 36 included studies had larger samples of males coinfected with cancer and HIV than females. There is evidence suggesting that men are more prone to developing cancer when compared with women, and while the reasons for this are multipronged, the primary difference is linked to the genetic or molecular level regulation including sex hormones like estrogen [80, 81].

### 4.1. Implications for Practice

This study presents a need for policy makers in public health to take note of the increasing trends of NADCs in the light of PLHIV living longer on cART. This will facilitate planning and decision making aimed at developing targeted effective interventions that can prevent or lead to early diagnosis and treatment of these cancers. The findings also suggest that the high rates of ADCs particularly in LMICs where some PLHIV are not on ART requires a swift response by each country not only to adopt the WHO “test and treat all policy” but to also implement it and evaluate its effectiveness. The policy was recommended in 2016 for people infected with HIV to start ART on the same day of diagnosis, and by June 2019, 93% of all the LMICs had adopted it while only 84% had started to put it in practice [37]. Evidence from this review further points to a need to consider male gender as an important predictor of cancer, and strategies of promoting early access to cancer care services by men may need to be considered. Finally, our study findings showed that there were limited data on ADCs and NADCs among PLHIV in LMICs especially SSA suggesting the need for future research in these settings.

### 4.2. Limitations

Our narrative review has some limitations. First, two-thirds of the studies used a retrospective design where the accuracy of data cannot be verified due to the use of records that were not designed for the study. Secondly, various methodologies in this review bring in differences in designs in the study settings, population, procedures, and data quality, making it difficult to make accurate comparisons. Thirdly, there was paucity of studies from LMICs, especially in SSA, thus making it difficult to generalize the findings to all the regions. However, notwithstanding these limitations, this review presents evidence that shows the increasing cancer trends in PLHIV, especially the NADCs, and supports the need for effective cancer screening strategies to reduce the cancer burden through early detection and appropriate treatment.

## 5. Conclusion

Chronic HIV infection combined with advancing age in PLHIV who are on cART are major contributors to the increasing cancer trends, particularly the incidence of NADCs, and the related mortality in this population. Our study provides evidence that when compared with the general population, PLHIV have an increased risk of both ADCs and NADCs with specific cancers such as lung, NHL, KS, cervical, and breast cancers being the most common. These findings stress the importance of screening for cancers among PLHIV, and the need for further research, to better understand the pathogenesis and predictors of both ADCs and NADCs and the increasing incidence of NADCs both virus and nonvirus-related NADCs especially in LMICs, to guide the development of targeted preventive and therapeutic interventions.

## Figures and Tables

**Table 1:** Characteristics of the included studies reporting cancer incidence and mortality.

Author & year	Study population		Study setting	Study design	Study period	Key findings				
						ADC studied	Most common ADC	NADC studied	Most common NADC	Mortality assessed
	Population type	Gender (those with cancer)								
Kauma et al., 2023 [40]	167 PLHIV & also with cancer	Female (58.1%) Male (41.9%)	Uganda (LIC)	Cross-sectional	2018-2019	Cervical, KS, and NHL	Cervical	Head & neck, esophageal, breast, & others	Breast	No
Muturi et al., 2023 [41]	301 with cancer, 32 (10.6%) PLHIV, and 269 (89.4%) HIV negative	Female (67.8%) Male (32.2%)	Kenya (LMIC)	Cross-sectional	2021	Cervical, KS, and NHL	Cervical	Prostate, lung, gastric, gall bladder, HL breast, tongue, larynx, anal, and head & neck	Breast	No
Arora et al., 2021 [42]	1258 PLHIV and 17 (1.4%) with cancer	Female (41.2%) Male (58.8%)	India (LMIC)	Retrospective & prospective cohorts	2011-2018	NHL, KS, and invasive cervical cancer	NHL	Lung, leukemia, breast, tongue, larynx, HL, and anal	HL	(i) Mortality rates were similar in both ADCs and NADCs (ii) Mean survival duration was 29 and 15 months for ADC and NADC, respectively
Horner et al., 2021 [43]	521623 PLHIV and 31611 (6.1%) with cancer	Gender not specified	USA (HIC)	Retrospective cohort	2001-2015	Cervical, KS, and NHL	NHL	Anus, liver, HL, breast, prostate, lung, & colorectal	Lung	Mortality rate was 386.9 & highest in males (401.4) versus 348.1 in females (i) Leading with high cancer-attributable mortality rates were NHL (92.6) and lung (63.0) (ii) Cancer-attributable mortality was highest among those aged ≥ 60 years (iii) Mortality declined by years: 484/100000 PY in 2001-2005 to 313.6/100000 PY in 2011-2015
Mendoza et al., 2021 [44]	269 PLHIV & also with cancer (276 cancers)	Female (20.7%) Male (79.3%),	Peru (UMIC)	Cross-sectional	2000-2018	KS, NHL, & invasive cervical cancer	KS	HL, skin, & cervical cancer in situ	HL	No
Patel et al., 2021 [45]	6641 PLHIV and 543 (8.2%) with cancer	Female (21.7%) Male (78.7%)	USA (HIC)	Retrospective cohort	2005-2011	Cervical, KS, and NHL	NHL	Colorectal, lung, anal, renal pelvis, liver, prostate, HL multiple myeloma, bladder, & breast	Colorectal	No
Spence et al., 2021 [46]	7912 PLHIV and 706 (8.9%) with cancer	Female (19.7%) Male (80.3%)	USA (HIC)	Longitudinal observational cohort	2011-2017	NHL, cervical, and KS	NHL	Breast, prostate, skin, melanoma, lung, nonmelanoma, anal, liver, head/neck, colorectal, HL, & renal	Breast	No
Wang et al., 2021 [47]	438 PLHIV & also with cancer	Female (19%) Male (81%)	China (UMIC)	Retrospective cohort	2007-2020	NHL, KS, and cervical	NHL	Lung, thyroid, gastric, breast hepatic, rectal carcinoma, leukemia, lymphoma, & others	Lung	No
Altuntas et al., 2020 [48]	1872 PLHIV and 48 (2.6%) with cancer	Female (8.3%) Male (91.7%)	Turkey (UMIC)	Retrospective cohort	1998-2016	KS and NHL	KS	Gastrointestinal, urogenital, lung, laryngeal, & spinal cord	Not specified	More NADC deaths (53.8%) compared to ADC deaths (22.9%)
Calkins et al., 2020 [49]	236 PLHIV & also with cancer	Female (31%) Male (69%)	USA (HIC)	Retrospective cohort	1997-2014	NHL (the only ADC)	NHL	Lung, liver, HL, prostate, & breast	Lung	(i) Among 138 HIV-infected who died, 74% was attributed to cancer (ii) The HIV-infected with baseline CD4 < 200 had on average a 7-month shorter survival after cancer diagnosis than those HIV negative (iii) Women with HIV and CD4 < 200 had on average a 10-month shorter survival than women without HIV
Gheorghiță et al., 2019 [50]	110 PLHIV & also with cancer	Female (43.6%) Male (56.4%)	Romania (UMIC)	Observational & retrospective cohort	2010-2016	KS, NHL, & cervical	KS	Breast, colorectal, HL, anal, hepatocellular, bronchopulmonary, gastric, & others	Digestive & anal	Cumulative mortality is 15.9 for ADCs and 19.5 for NADCs with NHL leading ADC-related deaths
Sinha et al., 2019 [51]	999 PLHIV and 29 (2.9%) with cancer	Female (24.1%) Male (75.9%)	India (LMIC)	Cross-sectional	2013-2016	NHL & invasive cervical	NHL	HL, lung, gall bladder, skin, nasal, & colon	HL	No
Billa et al., 2018 [52]	1391 PLHIV & HCV coinfection and 94 (6.8%) with cancer	Gender not specified	France (HIC)	Prospective cohort	2005-2017	Individual ADCs not specified	Not specified	Lung & nonmelanin skin, non-hepatitis C virus liver-related cancers, & hepatitis C virus-related	Lung	No
Cornejo-Juarez et al., 2018 [53]	1126 PLHIV and 127 (11.3%) with cancer (NADCs only)	Female (20.5%) Male (79.5%)	Mexico (UMIC)	Observational & retrospective cohort	1990-2016	No	N/A	HL, anal, germinal, vulvovaginal, breast, & others	HL & vulvovaginal	High proportion of deaths were in germinal cancer, all *n* = 13 died
Fink et al., 2018 [54]	15869 PLHIV and 783 (4.9%) with cancer	Female (18%) Male (82%)	Argentina, Brazil, and Mexico (UMICs), Chile (HIC), and Honduras (LMIC	Retrospective cohort	2000-2015	KS, NHL, & cervical	KS	Anal, breast, colon, HL, lung, prostate, renal, skin, testicular, & others	Anal	(i) 231/783 (30%) died (ii) Survival in year 1 lower for ADCs & higher for NADC
Grover et al., 2018 [55]	81865 PLHIV and 814 (0.9%) with cancer	Female (4%) Male (96%)	USA & Canada (HIC)	Retrospective cohort	2000-2009	Invasive cervical	Cervical	Lung, anal oropharynx, & HL	Lung	(i) 483/814 = 59.3% died (ii) Lung cancer was leading mortality with 339 deaths
Ignacio et al., 2018 [11]	1137 with cancer, 257 (22.6%) PLHIV, and 548 (48.2%) HIV negative	Female (55.5%) Male (45.5%)	Uganda (LIC)	Cross-sectional	2015	Cervical, KS, and NHL	Cervical	Breast, esophageal, head & neck, colorectal, & prostate	Breast	No
Kowalkowski et al., 2014 [56]	31576 PLHIV, 967 (3.1%) with cancer (virus-related NADCs only)	Males only.	USA (HIC)	Retrospective cohort	1985-2010	No	N/A	Hepatocellular, anus, and HL	Hepatocellular	No
Nagata et al., 2018 [57]	1001 PLHIV and 61 (6.1%) with cancer (NADCs only)	Gender not specified	Japan (HIC)	Retrospective cohort	1997-2015	No	N/A	Liver, colorectal, colon, anorectal, gastric, lung, HL, & others	Liver	No
Park et al., 2018 [58]	4169 (9.8%) cancers in 42441 PLHIV and 7879 (7.5%) cancers in 104712 HIV negative	Gender not specified	USA (HIC)	Prospective cohort	1999-2015	NHL & KS	NHL	Anal, liver, lung, prostate, HL, HPV-related cancers, & others	Liver & prostate	No
Chiu et al., 2017 [31]	4918 PLHIV, 145 (2.9%) NADMs, & 123 (2.5%) ADMs	Female (11%) Male (89%)	Canada (HIC)	Retrospective cohort	1996-2008	Not specified	N/A	Lung, anal, breast, head/neck, prostate, liver, rectal, and renal	Lung	No
Engels et al., 2017 [59]	46956 PLHIV and 1997 (4.3%) with cancer	Gender not specified	USA & Canada (HIC)	Retrospective cohort	1995-2009	Cervical, KS, and NHL	NHL	Lung, anal, liver, & anal	Lung	(i) Deaths attributable to NADCs (7.1%) versus ADCs (2.6%) (ii) Over 50% of these deaths were attributable to NHL, lung, and liver cancers (iii) Mortality rate = 327/100000 PY of PWHIV higher than US general population during 2014 (186/100 000 PY)
Campbell et al., 2016 [60]	1127 PLHIV & also with cancer (NADCs only)	Female (36.4%) Male (63.6%),	Tanzania (LMIC)	Retrospective cohort	2002-2014	No	N/A	Lung, liver, and head & neck	Head & neck	No
Mayor et al., 2016 [61]	4213 PLHIV and 281 (6.7%) with cancer	Female (28%) Male (72%)	Puerto Rico (HIC)	Retrospective cohort	1992-2010	KS, NHL, & cervical	KS	Oro/pharynx, lung, bronchus, liver, anus colon/rectum, vagina, & others	Oropharynx	(i) Around 50% died within the first year of cancer diagnoses (ii) Lung cancer was leading in 1-year mortality
Salters et al., 2016 [62]	2211 PLHIV and 77 (3.5%) with cancer	Females only (*n* = 77)	Canada (HIC)	Retrospective cohort	1994-2008	Cervical, NHL, and KS	Cervical	Breast, respiratory system, HL, & digestive system	Respiratory system	No
Yang et al., 2016 [63]	1946 PLHIV and 149 (7.7%) with cancer	Female (26.8%) Male (73.2%)	China (UMIC)	Retrospective cohort	2008-2013	NHL, KS, and cervical	NHL	Gastrointestinal, liver, HL, lung, & breast	HL	(i) Out of 149, 42 (28.2%) died, mortality rate = 0.78/100 PY (ii) More males (29/42 = 69%) versus females died (iii) Mortality rate for ADCs was higher (0.86) than NADCs (0.73)
Jaquet et al., 2015 [64]	1644 with cancer, 184 (11.2%) PLHIV, and 1460 (88.8%) HIV negative	Female (60.3%) Male (39.7%)	Benin, Côte d'Ivoire, and Nigeria (LMICs) and Togo (LIC)	Cross-sectional	2009-2012	Cervical, KS, and NHL	Cervical	Breast, liver, prostate, leukemia, colorectal, oropharynx, anogenital, & others	Breast	No
Raffetti et al., 2015 [65]	16268 PLHIV and 1159 (7.1%) with cancer	Female (21.6%) Male (78.4%)	Italy (HIC)	Retrospective cohort	1986-2012	KS, NHL, & cervical	KS	Liver, HL, colon, lung, rectum/anal, testes, larynx, penis, & others	Liver	(i) Annual standard mortality ratio (ASMR) for ADCs before and after 1998 decreased 2-3-fold from 96.1 to 29.1 (ii) ASMR for NADCs increased 2-fold from 14.3 to 27.5 around same period
Silverberg et al., 2015 [66]	86620 PLHIV and 196987 HIV negative	Gender not specified	USA & Canada (HIC)	Prospective cohort	1996-2009	KS and NHL	KS	Lung, anal, liver, HL colorectal, melanoma, oropharynx, & others	Lung	No
Gotti et al., 2014 [32]	13388 PLHIV and 866 (6.5%) with cancer	Female (22%) Male (78%)	Italy (HIC)	Retrospective cohort	1998-2012	KS, NHL, & cervical	NHL	Liver, HL, lung, & breast	Liver	No
Calabresi et al., 2013 [67]	5090 HIV-infected and 390 (7.7%) with cancer	Female (19.2%) Male (80.8%)	Italy (HIC)	Retrospective cohort	1999-2009	KS, NHL, & cervical	KS	HL, liver, melanoma, skin nonmelanin, trachea/lung, prostate, testes, & others	Virus related: liver Nonvirus related: skin nonmelanin	No
Coghill et al., 2013 [68]	802 with cancer, 274 (34.2%) PLHIV, & 528 (65.8%) HIV negative	Gender not specified	Uganda (LIC)	Retrospective cohort	2003-2010	Cervical and NHL	Cervical	HL, breast, & esophageal	Breast in the HIV-infected and HL in the HIV-uninfected	(i) HIV-infected were more than twice as likely to die during the year following cancer diagnosis compared with HIV-uninfected cancer patients (ii) HIV-infected diagnosed with infection-related cancers had greater than 50% higher risk of death during the year following cancer diagnosis (iii) HIV-infected diagnosed with cancers without an infectious cause also experienced significantly higher risk of death
Yanik et al., 2013 [69]	11485 PLHIV and 457 (4%) with cancer	Gender not specified	USA (HIC)	Observational cohort	1996-2011	KS, NHL, & cervical	KS	Lung, anal, breast, liver prostate, HL, colorectal, melanoma, & others	Breast	No
Pinto et al., 2012 [70]	730 PLHIV and 30 (4.1%) with cancer	Female (33.3%) Male (66.7%)	Brazil (UMIC)	Cross-sectional	2010-2011	KS, cervical, & NHL	KS	Hepatocellular, prostate, lung, HL, laryngeal, renal, colon, & penis	Prostate	No
Achenbach et al., 2011 [71]	20,677 PLHIV and 650 (3.1%) with cancer	Female (14%) Male (86%)	USA) (HIC)	Multisite retro and prospective cohort	1996-2009	KS, cervical, & NHL	KS	Lung, anal, prostate, HL, breast, colorectal, liver, & others	Lung	(i) Crude mortality rate was 20.6/100 PY (ii) Highest mortality was seen in primary CNS NHL, liver, and lung cancers
Dauby et al., 2011 [72]	3126 PLHIV and 45 (0.03%) with cancer (NADCs only)	Female (33.3%) Male (66.7%)	Belgium (HIC)	Retrospective cohort	2002-2009	No	N/A	HL, anal, lung, hepatocellular, prostate, bladder, breast, head & neck, & others	Anal in men HL in women	Out of 45 diagnosed with cancer, 20 (44.4%) died

ADCs: AIDS-defining cancers; NADCs: non-AIDS-defining cancers; KS: Kaposi sarcoma; NHL: non-Hodgkin lymphoma; HL: Hodgkin lymphoma; SCCA: squamous cell carcinoma anus; HPV: human papillomavirus; ART: antiretroviral therapy; HAART: highly active antiretroviral therapy; PLHIV: people living with HIV; ASMR: adjusted standardized mortality rate; PY: person years; HIC: high-income country; UMIC: upper-middle income country; LMIC: lower-middle income country; LIC: low-income country.

**Table 2:** Incidence and mortality trends for ADCs and NADCs in PLHIV and HIV-negative subgroups.

Article	Country	Incidence		Comparison between PLHIV & HIV negatives	Mortality		Comparison between PLHIV & HIV negatives	Incidence		Mortality	
		ADCs	NADCs		ADCs	NADCs		Virus-rel. NADCs	Nonvirus-rel. NADCs	Virus-rel. NADCs	Nonvirus-rel. NADCs
Achenbach et al., 2011 [71]	USA	˅	˄	X	X	X	X	˅	˄	˅	˄
Altuntas et al., 2020 [48]	Turkey	˄	˅	Increased risk of KS and urogenital cancers in PLHIV versus HIV negative	˅	˄	X	X	X	X	X
Arora et al., 2021 [42]	India	˅	˄	X	~	~	X	X	X	X	X
Billa et al., 2018 [52]	France	˅	˄	X	X	X	X	X	X	X	X
Calabresi et al., 2013 [67]	Italy	˄	˅	Overall cancer risk 4-fold higher in PLHIV than HIV negative	X	X	X	˄	˅	X	X
Calkins et al., 2020 [49]	USA	˅	˄	X	X	X	Low CD4 < 200 associated with shorter survival in PLHIV especially women	X	X	X	X
Chiu et al., 2017 [31]	Canada	˅	˄	Higher standard incidence rate for development of NADCs in PLHIV versus HIV negative	X	X	x	X	X	X	X
Coghill et al., 2013 [68]	Uganda	˄	˅	(i) More ADCs (cervical and NHL) diagnosed in PLHIV versus HIV negative (ii) More NADCs (breast and esophageal) diagnosed in HIV negative versus PLHIV	X	X	Risk of death twice for HIV pos 1-year after cancer diagnosis	X	X	X	X
Engels et al., 2017 [59]	USA & Canada	˅	˄	X	˅	˄	Mortality rate of PLHIV higher than US general population in 2014	X	X	X	X
Fink et al., 2018 [54]	Argentina, Brazil, Mexico, Chile, & Honduras	˄	˅	X	^∗^ ˄	˅	X	X	X	X	X
Gheorghiță et al., 2019 [50]	Romania	˄	˅	X	˅	˄	X	X	X	X	X
Gotti et al., 2014 [32]	Italy	~	~	X	˅	˄	X	X	X	X	X
Grover et al., 2018 [55]	USA & Canada	˅	˄	X	˅	˄	X	X	X	X	X
Horner et al., 2021 [43]	USA	˅	˄	X	˄	˅	X	X	X	X	X
Ignacio et al., 2018 [11]	Uganda	˅	˄	X	X	X	X	X	X	X	X
Jaquet et al., 2015 [64]	Benin, Côte d'Ivoire, Nigeria, & Togo	˅	˄	X	X	X	X	X	X	X	X
Kauma et al., 2023 [40]	Uganda	~	~	X	X	X	X	X	X	X	X
Mayor et al., 2016 [61]	Puerto Rico	˅	˄	(i) The incidence rate of ADCs in the late ART era was 17 times higher in PLHIV than the general population (ii) Incidence rate of NADCs in late ART era remained twice higher in PLHIV versus general population	^∗^ ˅	˄	X	X	X	X	X
Mendoza et al., 2021 [44]	Peru	˄	˅	X	X	X	X	X	X	X	X
Muturi et al., 2023 [41]	Kenya	˅	˄	X	X	X	X	X	X	X	X
Park et al., 2018 [58]	USA	˅	˄	(i) More ADCs in PLHIV versus HIV negative (ii) More virus-related NADCs in PLHIV versus HIV negative (iii) More nonvirus-related NADCs in HIV negative versus PLHIV	X	X	X	˅	˄	X	X
Patel et al., 2021 [45]	USA	˅	˄	X	X	X	X	X	X	X	X
Pinto et al., 2012 [70]	Brazil	˄	˅	X	X	X	X	X	X	X	X
Raffetti et al., 2015 [65]	Italy	˄	˅	(i) Overall incidence of ADCs was higher in PLHIV than the Italian general population (ii) Incidence of NADCs in PLHIV was similar to that of the Italian general population	^∗∗^^˄^ ˅	˅ ^˄^	X	X	X	X	X
Salters et al., 2016 [62]	Canada	˄	˅	HIV positive women compared to the general population were more likely to be diagnosed with cervical cancer, HL, NHL, & KS	X	X	X	X	X	X	X
Silverberg et al., 2015 [66]	USA & Canada	˅	˄	Cumulative cancer incidence by age 65 and 75 years during 1996–2009 was higher in PLHIV versus HIV negative for all cancer types except colorectal, melanoma, and oropharynx	X	X	X	X	X	X	X
Sinha et al., 2019 [51]	India	˄	˅	X	X	X	X	X	X	X	X
Spence et al., 2021 [46]	USA	˅	˄	X	X	X	X	X	X	X	X
Wang et al., 2021 [47]	China	˄	˅	X	X	X	X	X	X	X	X
Yang et al., 2016 [63]	China	˅	˄	X	˄	˅	X	X	X	X	X
Yanik et al., 2013 [69]	USA	˄	˅	X	X	X	X	˅	˄	X	X

Key: ˄ = higher rate; ˅ = lower rate, ~ = similar rate, X = not part of the study. ^∗^Mortality rate in the first year of ART. ^∗∗^ADC mortality higher in pre-ART and lower post-ART while NADC lower in pre-ART and higher post-ART. ADCs: AIDS-defining cancers; NADCs: non-AIDS-defining cancers; HIV: human immunodeficiency virus; KS: Kaposi sarcoma; NHL: non-Hodgkin lymphoma; HL: Hodgkin lymphoma; ART: antiretroviral therapy; HAART: highly active antiretroviral therapy; PLHIV: people living with HIV.

**Table 3:** Risk factors associated with cancer incidence and mortality among PLHIV.

Risks for cancer incidence (*N* = 15 studies)	HIV infection/longer duration with HIV infection	Opportunistic infections	Increasing age/age > 45 years/age > 50 years	Low CD4/CD4 < 50/CD4 < 200	Lack of viral load suppression	Male gender	Smoking/tobacco use	Clinical AIDS comorbidity	Cancer history	Absence of ART
Any cancer	2 (13.3%)	—	3 (20%)	2 (13.3%)	—	1 (6.7%)	1 (6.7%)	1 (6.7%)	—	1 (6.7%)
ADCs	—	1 (6.7%)^∗^	—	3 (20.0%)	1 (6.7%)	—	—	—	1 (6.7%)^∗^	—
NADCs	2 (13.3%)	—	5 (33.3%)	3 (20.0%)	—	1 (6.7%)	1 (6.7%)	—	—	1 (6.7%)
Risks for cancer mortality (*N* = 7 studies)	Poor immune status	Lack of cancer treatment	Cancer staging 3 or 4/cancer progression/relapse	Increasing age/age > 45/>50/>55 years	Nadir CD4 < 200/CD4 < 100	Failure to suppress HIV RNA/viral load > 400	Time since HIV infection (>5 years)	Previous AIDS/AIDS comorbidity	Not on ART	Male gender
Any cancer	1 (14.2%) ^∗^	1 (14.2%)^∗^	1 (14.2%)^∗^	2 (28.6%)	—	2 (28.6%)	1 (14.2%)		1 (14.2%)	
ADCs	—	—	—	—	2 (28.6%)	—	—	—	—	1 (14.2%)
NADCs	—	—	1 (14.2%)	2 (28.6%)	2 (28.6%)	—	1 (14.2%)	2 (28.6%)	—	—

^∗^Same study. PLHIV: people living with HIV; HIV: human immunodeficiency virus; RNA: ribonucleic acid; ADCs: AIDS-defining cancers; NADCs: non-AIDS-defining cancers; ART: antiretroviral therapy.

## Data Availability

The data reported and supporting this paper was sourced from the existing literature and is therefore available through the detailed reference list.
